# An Anxious Heart: The Relation Between Cardiovascular Disease and Prevalence of Anxiety Symptoms

**DOI:** 10.1192/j.eurpsy.2022.1764

**Published:** 2022-09-01

**Authors:** R. De Sousa, C. Solis, I. Silva, R. Gonçalves

**Affiliations:** 1 ACeS Baixo Mondego, Usf Coimbra Centro, Coimbra, Portugal; 2 ACeS Baixo Mondego, Usf Coimbra Centro, Apt º Esq, Portugal; 3 University of Coimbra, Medical School, Coimbra, Portugal

**Keywords:** diabetes, Cardiovascular, risk factors, Anxiety

## Abstract

**Introduction:**

Cardiovascular disease represent the leading cause of death worldwide, and is also responsible for the consumption of many medical resources, work absenteeism and worse Quality of Life. On the other hand, psychiatric diseases have recently gained more relevance worldwide as one of the principal causes of disability.

**Objectives:**

Evaluate a possible relationship between cardiovascular risk factors (CVRF) and anxious or depressive symptoms.

**Methods:**

Observational and cross-sectional study in a non-probabilistic and convenience sample, composed by patients followed on five primary healthcare facilites, who voluntarily accepted to answer the questionnaire through an interview, between July 2020 and January 2021. After an informed consent, a questionnaire was carried out including sociodemographic characterization, presence of cardiovascular disease and/or cardiovascular risk factors and the Portuguese version of HADS. Descriptive and inferential statistics were performed, using Mann-Whitney U test. A value of p<0,05 was considered statistically significant.

**Results:**

Sample of 179 people, 53,1% female, with an average age of 51,05 ± 22,02 years, in which 57,5% had one or more CVRF and 59,8% had CVD and/or CVFR and the most prevalent CVRF were hypertension (48%) and dyslipidemia (43,6%). There was a statistically significant relationship between diabetes and anxiety (p<0,05).

**Conclusions:**

There was a greater prevalence of anxiety symptoms in people with diabetes compared to people without diabetes. This suggests the importance of giving attention to anxiety in patients with diabetes, given the relevance of this comorbidity in their quality of life. The main limitation of the study is related with the sample size.

**Disclosure:**

No significant relationships.

